# Therapeutic Potential of Astragaloside IV Against Adriamycin-Induced Renal Damage in Rats *via* Ferroptosis

**DOI:** 10.3389/fphar.2022.812594

**Published:** 2022-03-18

**Authors:** Lu-Yun Qin, Peng Guan, Jian-Xin Wang, Yu Chen, Ya-Shuo Zhao, Sheng-Chang Yang, Ya-Jing Guo, Na Wang, En-Sheng Ji

**Affiliations:** ^1^Department of Physiology, Hebei University of Chinese Medicine, Shijiazhuang, China; ^2^College of Life Science, Hebei Normal University, Shijiazhuang, China; ^3^Hebei Technology Innovation Center of TCM Formula Preparations, Shijiazhuang, China

**Keywords:** kidney, astragaloside Ⅳ, Adriamycin, iron metabolism, ferroptosis

## Abstract

Adriamycin (ADR) has been utilized to treat cancer for several decades. However, ADR-induced renal injury is one of the most common side effects accompanying ADR therapy. In the present study, we revealed that astragaloside IV (ASIV) was beneficial for renal injury caused by Adriamycin. We demonstrated that ASIV significantly ameliorated kidney injury, improved renal dysfunction, reduced oxidative stress, alleviated iron accumulation, and inhibited the induction of ferroptosis by ADR. ASIV also rescued the intracellular levels of nuclear factor-erythroid-2-related factor 2 (Nrf2) and promoted nuclear translocation of Nrf2. These protective effects of ASIV on renal injury might be attained through the ASIV-induced activation of the Pi3K/Akt signaling pathway. *In vitro*, the treatment of the HK-2 cells with fer-1 or deferoxamine mesylate obviously improved cell viability during Adriamycin administration. On the other hand, the protective role of ASIV can be abrogated by RSL3 to some extent. Moreover, ASIV lowered the expression of transferrin receptor 1 and divalent metal transporter 1 while enhancing the expression of ferropotin 1 and glutathione peroxidase 4 in ADR administrated cells, the effects of which were akin to those of deferoxamine mesylate. Furthermore, ASIV increased the phosphorylation of Pi3K, Akt, and the expression of Nrf2 and glutathione peroxidase 4 compared to HK-2 cells stimulated by ADR. However, Pi3K inhibitor LY294002 abrogated these activations. In conclusion, ferroptosis may involve in ADR-induced nephrotoxicity, and ASIV might protect nephrocytes against ADR-induced ferroptosis, perhaps *via* activations of the Pi3K/Akt and Nrf2 signaling pathways.

## Introduction

Adriamycin (ADR) has been widely used for treatment against malignant tumors. However, the utilization of ADR is limited by its adverse effect that leads to renal, cardiac, and testicular toxicity (Rajagopalan et al., 1988; Takemura and Fujiwara, 2007; Albini et al., 2010; Fukasawa et al., 2014). Burke et al. (Burke et al., 1977) first described a case of a 78-year-old man developing renal failure after the administration of Adriamycin in 1977. Then the nephrotoxicity of Adriamycin has been widely used to stimulate chronic proteinuric renal disease in humans as an experimental rodent model of kidney disease (Lee and Harris, 2011). Although the exact mechanism of Adriamycin-induced nephrotoxicity remains unknown, it is believed that the toxicity may be mediated through free radical formation, iron-dependent oxidative damage of biological macromolecules, membrane lipid peroxidation, and protein oxidation (Liu et al., 2007). Additionally, the administration of ADR yielded significant iron deposition in proximal tubular (Van den Branden et al., 2002). Since ferroptosis is characterized by iron-dependent lipid peroxidation on cell membranes, research has to be carried out to find whether ferroptosis is the initial trigger in ADR-induced renal injury. Meanwhile, understanding the mechanisms will help find protective strategies for chemotherapy patients.

Transition metal iron is a crucial element for almost all living organisms because it is needed to transport oxygen, produce energy, and participate in many metabolic processes (Pantopoulos et al., 2012). Although essential for living, excess iron can generate toxic reactive oxygen species (ROS) (Wang et al., 2018). Ferroptosis is characterized by iron-dependent increase in lipid peroxides, which can inhibit the activities of system glutathione peroxidase 4 (GPx4) and solute carrier family 7 member 11 (SLC7A11) (Cao and Dixon, 2016). Ferroptosis has recently gained much attention in diverse kidney diseases as emerging evidence shows that ferroptosis participates in the pathogenesis of kidney diseases, such as diabetic nephropathy (Li et al., 2021) and rhabdomyolysis-related kidney damage (Guerrero–Hue et al., 2019). GPx4 is a GSH-dependent enzyme that reduces lipid hydroperoxides (L-OOH), producing oxidized glutathione (GSSG), and negatively regulates ferroptosis (Seiler et al., 2008). In this sense, pharmacological GPx4 activators may be used to prevent ferroptosis-induced tissue *in vivo* (Friedmann Angeli et al., 2014). Consistent with this view, mice with induced deletion of GPx4 died of massive cell death of renal tubular epithelia within 2 weeks (Friedmann Angeli et al., 2014). However, the impact of ferroptosis on the development of ADR-induced renal damage has not been elucidated.

Diverse ROS scavengers have been shown to alleviate Adriamycin-induced nephrotoxicity in rats (Liu et al., 2018; Amarasiri et al., 2020; Huang et al., 2022). Astragaloside IV (ASIV) has been found to inhibit Adriamycin-induced ferroptosis in the heart in addition to the antioxidant function in our previous research (Luo et al., 2021a). We noticed that inhibition of lipid peroxidation and protection against GSH depletion are beneficial to limiting Adriamycin toxicity in the kidney (El-Shitany et al., 2008; Nazmi et al., 2011). Thus, ASIV was chosen to investigate its effect on the nephrotoxic potential of Adriamycin.

The previous research has demonstrated that Pi3K/Akt activation is essential for Nrf2 to impart protective effects in kidney diseases (Zhang et al., 2021). We hypothesized that ASIV may play a protective role against ferroptosis in ADR-induced renal injury, perhaps through the Pi3K/Akt and Nrf2 pathways. To test our hypothesis, we conducted a series of histological and molecular experiments to determine the regulatory mechanism of ASIV in ADR-induced kidney damage, aiming to provide a novel protective approach for chemotherapy patients.

## Materials and Methods

### Animal Procedure

Male SD rats (200 ± 10 g) were obtained from Beijing Vital River Company (Beijing, China). They were kept under 12-h light/dark cycles and allowed free access to food and water. ASIV (No. M0502A) was purchased from Meilunbio (Dalian, China). Rats were randomly divided into CON, ADR, ADR + ASIV, and ASIV groups (*n* = 6). Rats in ADR and ADR + ASIV groups received four equal injections of ADR intraperitoneally (4 mg/kg) in 5 weeks. Rats in ASIV and ADR + ASIV groups intragastrically received ASIV (10 mg/kg, daily) for 5 weeks, while rats in CON and ADR groups were administered the same dose of solvent as ADR. Finally, the rats were euthanized, and the bilateral kidneys were excised. The animal study was reviewed and approved by the Animal Care and Use Committee of Hebei University of Chinese Medicine.

### Cell Culture

HK-2, a proximal tubular cell line derived from a normal kidney, were obtained from frozen storage in our laboratory. The cells were maintained in commercially available Dulbecco’s modified Eagle medium/Nutrient Mixture F-12 (DMEM/F-12) medium. The culture medium was supplemented with 10% fetal bovine serum (FBS, Cat. No. 10270-106), 100 U/mL penicillin, and 100 μg/ml streptomycin. The cells were seeded and left to grow to 80% confluence, and then transferred to a serum-free medium for 12 h prior to each experiment. Finally, the cells were incubated with ADR (1 μM; A0702A) in the presence or absence of ASIV (100 μM; M0502A), Pi3K inhibitor LY294002 (20 µM), iron chelator deferoxamine mesylate (DFO; 30 µM), ferroptosis inducer RSL3 (1 μM, s8155), and ferroptosis inhibitor ferrostatin-1 (Fer-1, 3 µM).

### Cell Viability Assay

According to the manufacturer’s instructions, Cell Counting Kit-8 (CCK-8, ApexBio, Cat No: K1018) was used to detect cell viability. In brief, HK-2 cells were seeded in 96-well plates and treated as discussed earlier. 10 µL of CCK-8 solution was added to each well of the plate, incubating with the cells at 37°C for 2 h. A microplate reader (Thermo Fisher, Waltham, MA, United States) was used to measure the optical density value at the wavelength of 450 nm.

### sCR and BUN Measurement

The serum creatinine (sCR) and serum urea nitrogen (BUN) levels were used to evaluate the renal function. Blood was drawn from each rat after being sacrificed. Following centrifugation at 1,500 rpm for 15 min, serum was isolated and was stored at −20°C until usage. sCR and BUN levels were determined with a sCR detection kit (Nanjing Jiancheng Bioengineering Institute, China, C011-2) and a BUN detection kit (Nanjing Jiancheng Bioengineering Institute, China, C013-2), respectively.

### Histopathological Examination

Following excision, the kidney was immersed in 4% paraformaldehyde for 2 days at 4°C. Tissues were then dehydrated stepwise in ethanol, cleared with dehydrated xylene, embedded in paraffin, sliced into 4-μm-thick sections, and were deposited on glass slides. Hematoxylin and eosin (H&E) staining was used to obtain the pathological condition. The histological slides of the kidney were evaluated for semiquantitative analysis, as Raij L et al. described previously (Raij L et al., 1984). Masson’s trichrome staining was used to detect the collagen fibers in the kidney. Immunohistochemistry was employed with an anti-GPx4 antibody (1:200, Cat#ET1706-45, HUABIO) and was utilized, according to the manufacturer’s instructions. The intensities of GPx4 in the photos were detected by ImageJ software.

### Perls’ Staining

Iron accumulation in the kidney was evaluated using enhanced Perls’ Prussian blue reaction. The slides were passed through xylene and the descending alcohols to deparaffinize and rehydrate. Each slide was incubated in 1% potassium ferrocyanide (w/v) in 1% HCl (v/v) for 1 h. Diaminobenzidine (DAB) intensification was performed according to the method of Lakhal-Littleton et al. (2015). The sections were examined by Leica DM2500M microscope (Leica Microsystems, Wetzlar, Germany) and quantified by ImageJ software.

### Assessment of Biochemical Indicators

Malondialdehyde (MDA) is a stable end product of lipid peroxidation, and it stands for oxidation. Glutathione peroxidase (GPx) and superoxide dismutase (SOD) are responsible for eliminating free radicals as an antioxidant defense system. The biochemical changes in the renal cortex were investigated using commercial kits (Jiancheng Institute of Biotechnology, Nanjing, China, cat. no. A003-4-1, A005-1, and A001-3-2) to measure the levels of MDA and the enzyme bioactivity of GPx and SOD. The Bicinchoninic Acid Protein Assay Kit (CWBio, Beijing, China) was used to detect the protein content. Parameters MDA, GPx, and SOD were recorded on a microplate reader at wavelengths of 530, 412, and 450 nm, respectively.

### Detection of Reactive Oxygen Species in the Kidney Tissue

Dihydroethidium staining was used to detect renal ROS. Upon oxidation, dihydroethidium is intercalated in DNA, showing bright fluorescent red. In short, the frozen tissues were cryosectioned at 10 μm thick, mounted onto glass slides, and incubated with 10 μM dihydroethidium (Molecular Probes, Eugene, OR, United States) for 1 h at room temperature. After sealing, the sections were examined by Leica DM2500M microscope (Leica Microsystems, Wetzlar, Germany). ImageJ software was used for quantitative analysis.

### Detection of Intracellular ROS

A dichlorofluorescin diacetate (DCFH-DA) assay was performed to measure the levels of intracellular ROS. 10 µM DCFH-DA was added to each well of the 6-well plate, incubating with HK-2 cells for 30 min at room temperature in dark. After being washed 3 times with PBS, the ROS content was measured using an inverted microscope (IX73, Olympus). ImageJ software was used for quantitative analysis.

### Transmission Electron Microscopy

To investigate whether or not ferroptosis is involved in the pathology, transmission electron microscopy was performed. In brief, the tissue samples from renal cortex were cut into 1 mm^3^ blocks, treated in 2.5% glutaraldehyde in 0.1 M phosphate buffer (pH 7.2) for at least 24 h, post-fixed in 1% osmium tetroxide, dehydrated in graded ethanol series, and then embedded in Epon 812. Thin sections were cut with a microtome (Leica EM UC6, Leica Microsystems, Wetzlar, Germany), stained with uranyl acetate and lead citrate, examined, and photographed under a transmission electron microscope (H-7600, Hitachi, Tokyo, Japan).

### Quantitative Real-Time PCR

Total renal cortical RNA was purified with TRIzol (Invitrogen^®^, Carlsbad, CA, United States). Complementary DNA was synthesized by the reverse transcription of mRNA using MMLV (RR047A, TaKaRa, Dalian, China). Quantitative PCR was performed using 2 × SYBR Pre mix Ex Taq (RR820A, TaKaRa, Dalian, China) in a real-time PCR system (Bio-Rad, CFX Connection, Hercules, United States). Relative mRNA expression was calculated using the 2^−ΔΔCt^ method. The primers used are Ptgs2: forward, 5′- ATG TTC GCA TTC TTT GCC CAG-3′ and reverse, 5′-TAC ACC TCT CCA CCG ATG AC-3′; GAPDH: forward, 5′-GAG TCA ACG GAT TTG GTC GT-3′, and reverse, 5′-GAC AAG CTT CCC GTT CTC AG-3′.

### Western Blotting

Renal cortex tissue samples were homogenized in RIPA lysis buffer, and the Bicinchoninic Acid Protein Assay Kit (Cwbiotech, Beijing, China) was used to measure protein levels, as previously described (Guan et al., 2019). 40 μg protein from renal homogenate was applied to each lane for separation on SDS–PAGE. Immunoblotting was performed on the separated protein and probe with the following primary antibodies overnight at 4°C: TfR1 (1:2,000, Cat#abs131442, absin), FTL (1:10,000, Cat#3549-1, EPITMICS), DMT1 (1:2,000, Cat#abs112967, absin), FPN1 (1:3,000, Cat#GTX100573, absin), NOX2 (1:2,000, Cat#BA2811, BOSTER), NOX4 (1:1,000, Cat#GB11347, servicebio), GPx4 (1:2,000, Cat#ET1706-45, HUABIO), SLC7A11 (1:1,000, Cat#DF12509, Affinity), Nrf2 (1:1,000, Cat#M200-3, MBL), HO-1 (1:2,000, Cat#GB12104, servicebio), p-Pi3K (Cat#AF3241, Affinity), Pi3K (Cat#AF6241, Affinity), p-Akt (Cat#66444-1-Ig, Proteintech), Akt (Cat#60203-2-Ig, Proteintech), β-actin (1:1,000, Cat#CW0096S, CWBIO), and Lamin B1 (1:1,500, Cat#SI17-06, HUABIO). The secondary antibodies were diluted at 1:1,000. The blots were imaged and analyzed applying enhanced chemiluminescent detection (Vilber Fusion FX5 Spectra, Paris, France). The proteins were normalized against β-actin.

### Statistical Analysis

Results were expressed as mean ± S.D. Statistical analyses were performed using SPSS 19.0 (IBM, New York, NY). Statistical significance was evaluated using analysis of variance (ANOVA), followed by the Tukey–Kramer *post hoc* test. Results with a value of *p* < 0.05 were regarded as statistically significant.

## Results

### Astragaloside IV Attenuates Adriamycin-Induced Functional and Structural Renal Damage

**FIGURE 1:**
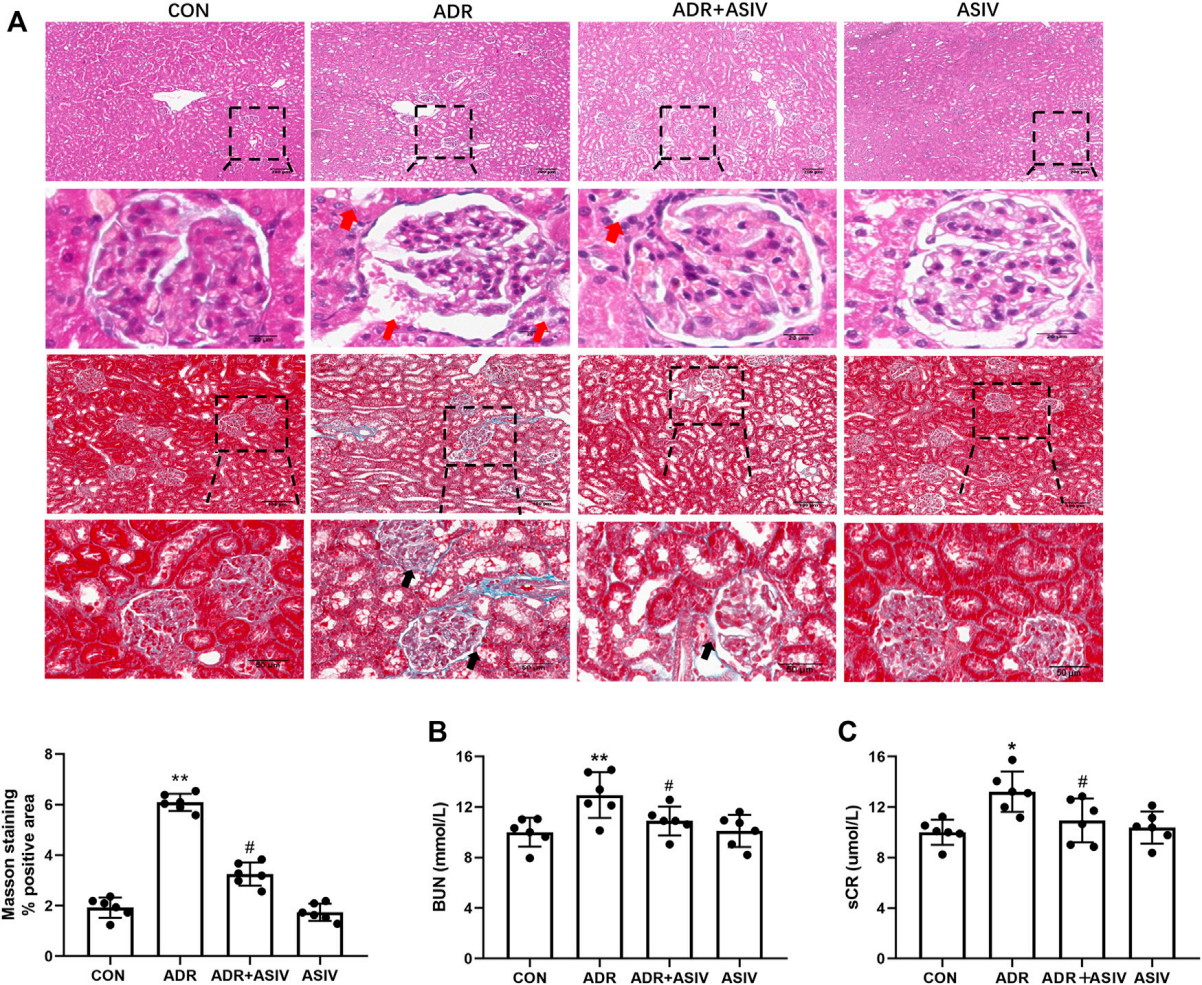
ASIV attenuates ADR-induced functional and structural renal damage. **(A)** Representative images of H&E and Masson staining of rat renal section in each group and quantification of fibrosis. Red arrows indicated atrophy of glomerulus and tubular lumen dilatation; black arrows indicated periglomerular fibrosis. **(B)** The levels of blood urea nitrogen (BUN) and **(C)** serum creatinine; data were expressed as mean ± SD, *n* = 6. *p < 0.05, **p < 0.01 vs. CON group. ^#^*p* < 0.05, ^##^*p* < 0.01 vs. ADR group.

H&E and Masson trichrome stain were performed to monitor renal histopathologic changes. The histological analysis showed that rats exposed to ADR had kidney damage, such as atrophy of glomerulus, tubular lumen dilatation, and periglomerular fibrosis (Figure 1A). In accordance, ADR-administrated rats showed elevated levels of blood urea nitrogen (BUN) and serum creatinine compared with the CON group (Figures 1B,C). These structural and functional changes were alleviated by ASIV, revealing a protective effect of this molecule against ADR-associated kidney injury.

### Astragaloside IV Reduced Oxidative Stress Associated With Adriamycin Nephropathy

**FIGURE 2:**
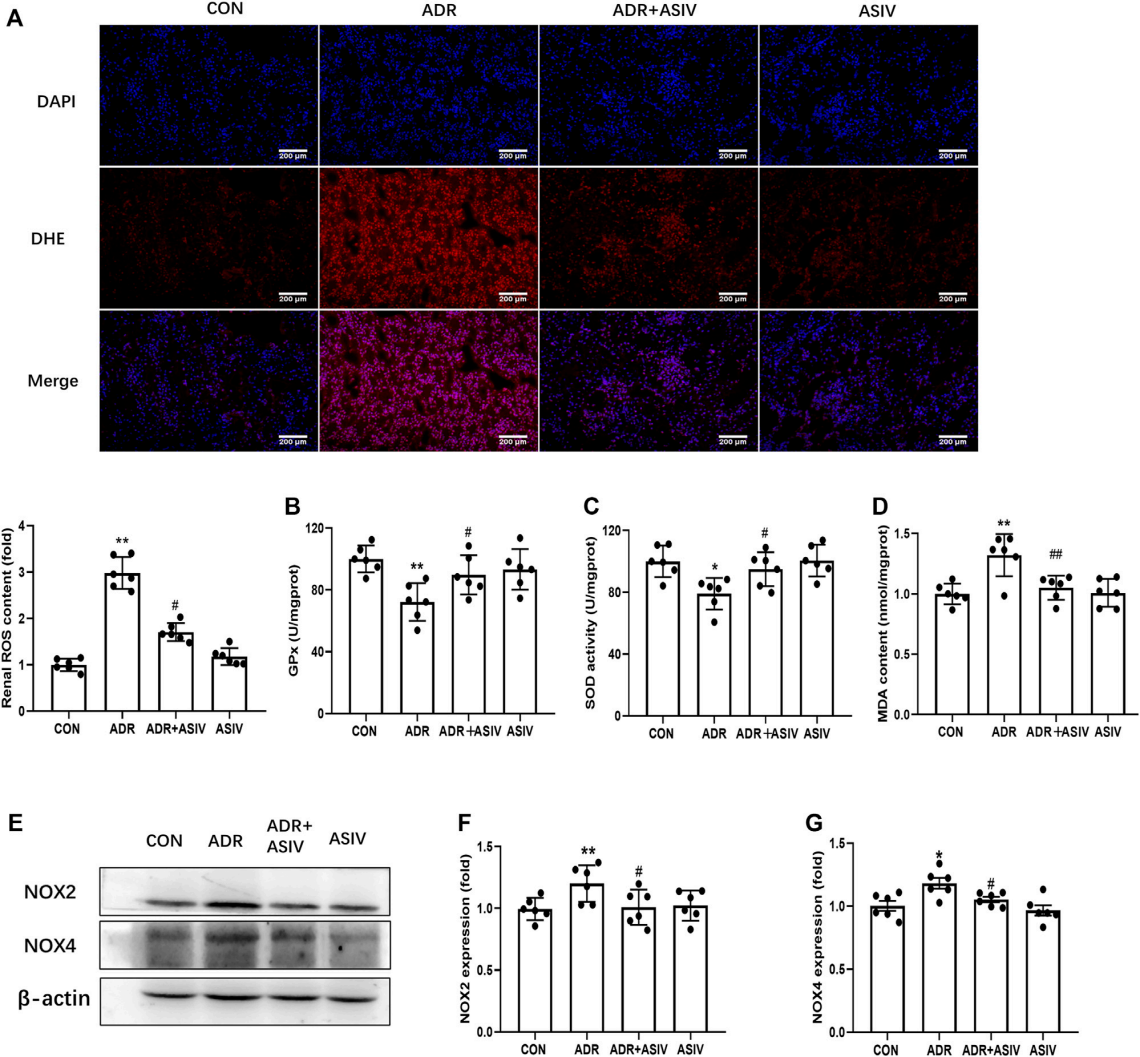
ASIV reduced ADR-induced oxidative stress. **(A)** Representative images of DHE staining and quantification of ROS production. DHE positive (red), nuclear stained with DAPI (blue). Serum GPx **(B)**, SOD **(C)**, and MDA **(D)** levels among the different groups (*n* = 3). **(E)** Protein expression of oxidative stress-associated proteins, including NOX2, NOX4 in kidney tissue. **(F,G)** The expression of NOX2 and NOX4. Data were expressed as mean ± SD, *n* = 6. *p < 0.05, **p < 0.01 vs. CON group. ^#^*p* < 0.05, ^##^*p* < 0.01 vs. ADR group.

Oxidative stress has been considered a key contributor to Adriamycin nephropathy (Karanovic et al., 2021). To explore the possibility that oxidative stress participated in ADR-induced renal injury, we examined some indexes about oxidative stress. In the ADR group, the generation of ROS was significantly induced compared with the CON group. As demonstrated in Figure 2A, ASIV treatment significantly decreased ROS production. The antioxidant enzyme activities of SOD and GPx were significantly decreased in the model group. In the ASIV-treated group, the activities of GPx and SOD were higher than those in the ADR group (Figures 2B,C). MDA, the lipid peroxidation product, was increased in the ADR group and was inhibited after ASIV administration (Figure 2D). NADPH oxidase (NOX) is a family containing seven enzymes that are involved in the generation of reactive oxygen species (ROS). ADR treatment increases the expression of NOX2 and NOX4 in the rat kidney, increasing oxidative stress leading to cardiomyopathy (McLaughlin et al., 2017). Therefore, we detected the levels of NOX2 and NOX4, and found that these proteins were significantly increased in the ADR group, while ASIV treatment reversed these changes (Figures 2E–G).

### Astragaloside IV Alleviated Iron Accumulation in Rats With Adriamycin-Induced Renal Damage

**FIGURE 3:**
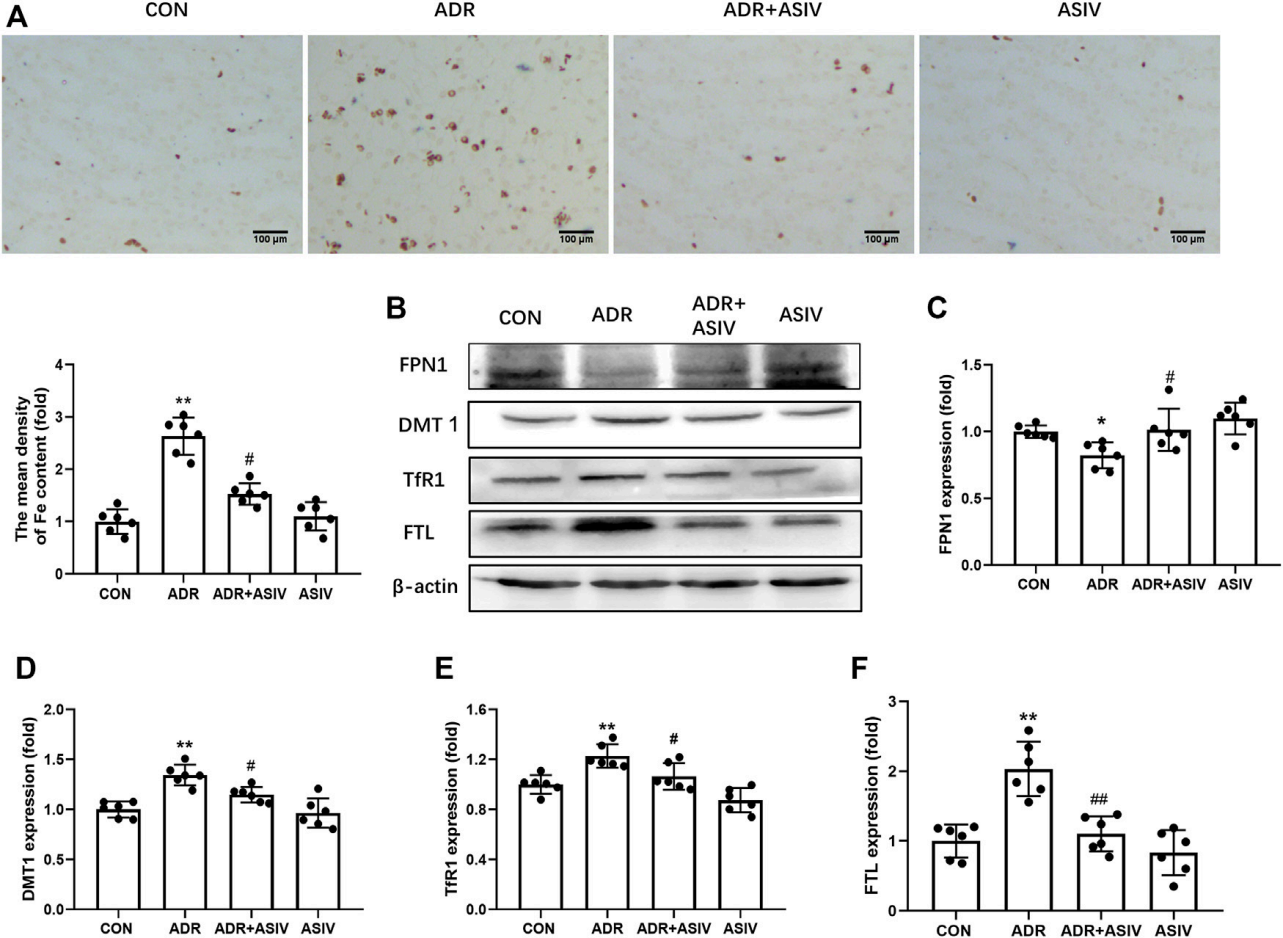
ASIV alleviated iron accumulation in rats with ADR-induced renal damage (ADR disrupts iron metabolism, which leads to iron deposition in renal tissue). **(A)** Representative images of Perls’ Prussian blue staining and quantification of iron accumulation. **(B)** Representative Western blotting photographs. **(C)** Renal FPN1 expression. **(D)** The expression of DMT1. **(E)** The expression of renal TfR1. **(F)** Renal FTL expression. Data were expressed as mean ± SD, *n* = 6. *p < 0.05, **p < 0.01 vs. CON group. ^#^*p* < 0.05, ^##^*p* < 0.01 vs. ADR group.

Given the role of iron in the generation of ROS and lipid peroxidation, we detected the iron content in kidney. Prussian blue staining showed that ADR administration induced the accumulation of renal iron significantly. However, this change was reversed by ASIV (Figure 3A). Iron metabolism associated proteins were detected by Western blot (Figure 3B). As shown in Figure 3C, the expression of iron exporting protein FPN1 was downregulated by ADR treatment. ASIV restored iron export by significantly increasing FPN1 expression compared to animals receiving only ADR. By contrast, the levels of DMT1 and TfR1 proteins were increased by ADR treatment, and ASIV inhibited its expression (Figures 3D,E). The expression level of FTL was upregulated in ADR-treated rats. ASIV significantly reduced the FTL protein level (Figure 3F).

### Astragaloside IV Alleviated Ferroptosis Induced by Adriamycin

**FIGURE 4:**
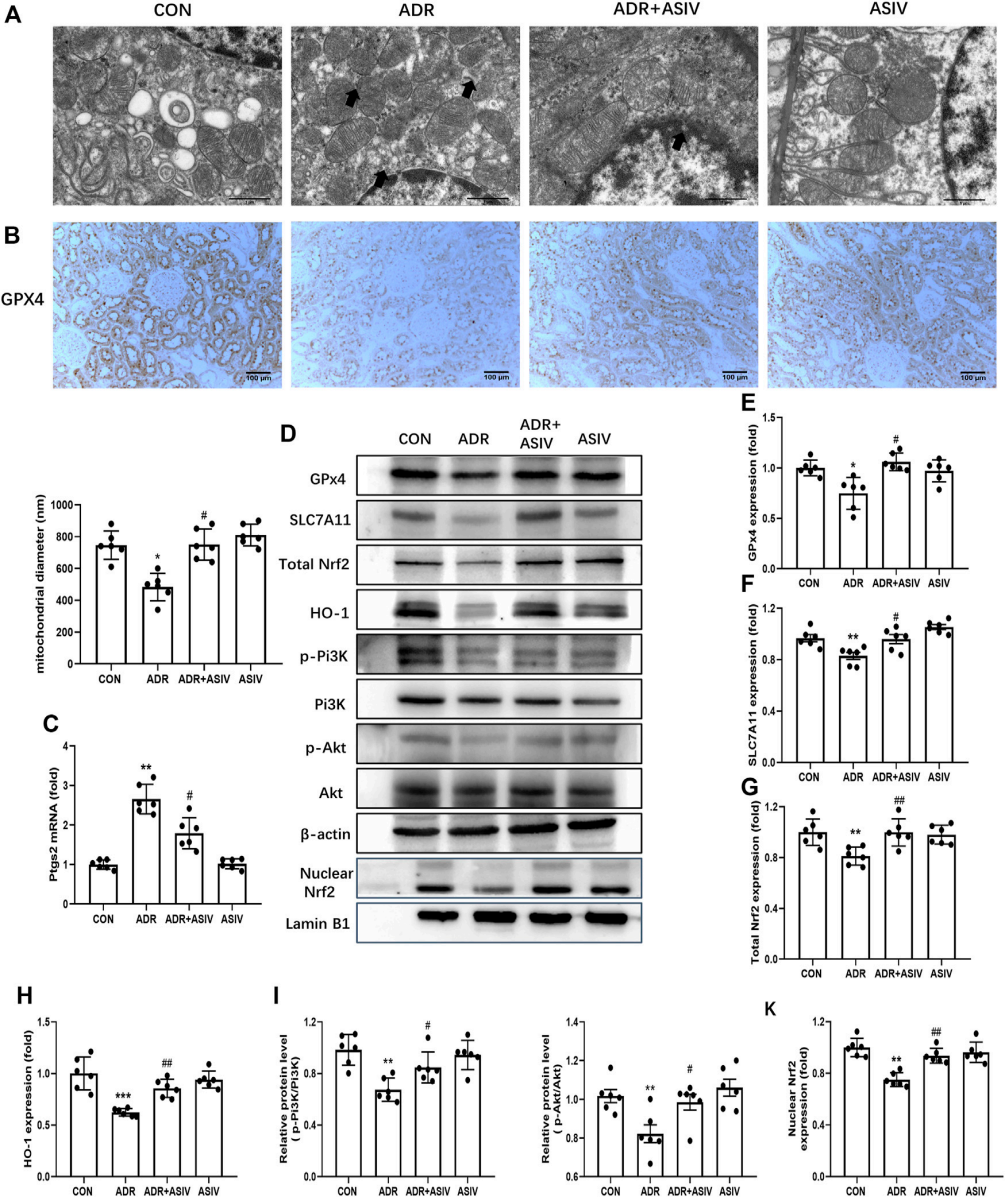
ASIV attenuated ferroptosis-related indicators in the ADR-reduced renal damage. **(A)** Representative TEM images demonstrating ADR-induced ferroptosis and quantification of mitochondrial diameters. Black arrows indicated disappeared mitochondrial ridge and outer membrane rupture. **(B)** Representative GPx4 immunohistochemical images. **(C)** The Ptgs2 mRNA levels in the renal. **(D)** Western blot for GPx4, SLC7A11, Nrf2, HO-1, p-Pi3K, and p-Akt in the rat kidney. **(E)** Quantification of GSH peroxidase 4 (GPx4). **(F)** Quantification of SLC7A11. **(G)** Quantification of total Nrf2. **(H)** Quantification of HO-1. **(I)** Quantification of p-Pi3K. **(J)** Quantification of p-Akt. **(K)** Quantification of nuclear Nrf2. Data were expressed as mean ± SD, *n* = 6. *p < 0.05, **p < 0.01 vs. CON group. ^#^*p* < 0.05, ^##^*p* < 0.01 vs. ADR group.

Ferroptosis was reported to exert a key role in the mechanism for the pathogenesis of renal injury (Hu et al., 2019). To study the mechanism underlying ADR-induced renal injury, ferroptosis in the kidney was detected. As shown in Figure 4A, the ultrastructural photos showed the morphological characteristics of ferroptosis in the ADR group, such as shrunken mitochondria, elevated membrane density, and disappeared mitochondria ridge. ASIV co-treatment considerably improved the ultrastructure of kidney in contrast to the ADR-alone group (Figure 4A). Consistent with ferroptosis, ADR induced a reduction of mitochondrial diameters in the ADR-alone group, which was blocked after ASIV was administrated. ASIV co-treatment increased the expression level of GPx4 compared with the ADR-alone treated group with immunohistochemistry analysis using anti-GPx4-antibody (Figure 4B). Upregulated ptgs2 mRNA indicated ADR induced ferroptosis onset (Figure 4C). The levels of some molecules involved in ferroptosis are shown in Figure 4D; the transportation of Nrf2 to the nucleus is shown in Figure 4K. As shown in Figures 4E,F, ADR treatment notably suppressed GPx4 and SLC7A11 expression. However, ASIV could restore the expression of both proteins, suggesting that ASIV played a protective role in ADR-induced kidney damage *via* inhibiting ferroptosis. To further investigate whether Pi3K/Akt and Nrf2 pathways were involved in ferroptosis, Western blotting showed that ASIV co-treatment obviously increased the expression of Nrf2 and HO-1, and the phosphorylation of Pi3K and Akt, which were inhibited by ADR alone (Figures 4G–J). ASIV also restored the nuclear translocation of Nrf2 (Figure 4K), indicating the involvement of Nrf2 transcription factor activation.

### Astragaloside IV Alleviated Ferroptosis Induced by Adriamycin *In Vitro*

**FIGURE 5:**
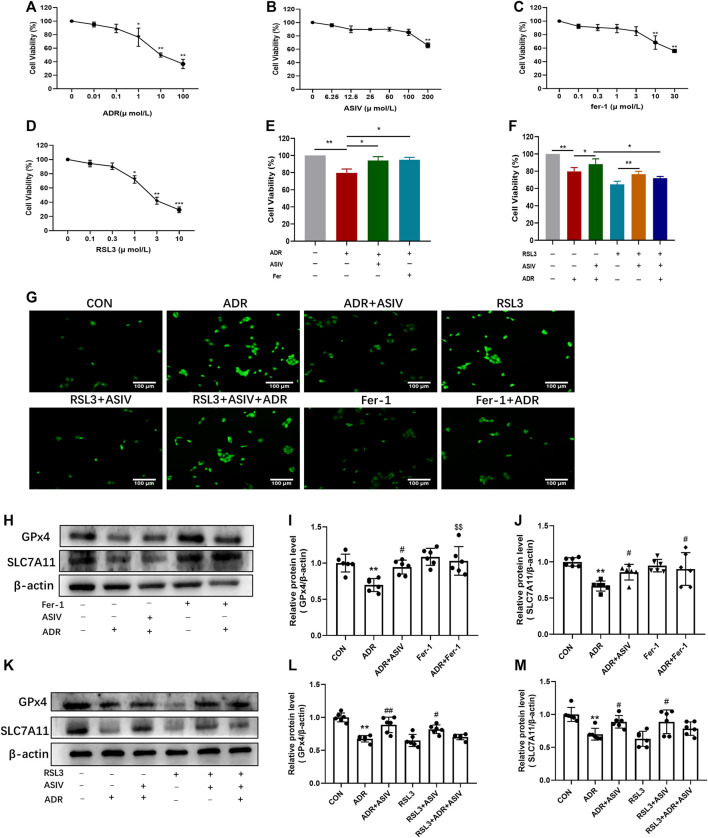
ASIV alleviated ADR-induced ferroptosis was associated with the inhibition of the Pi3K/Akt and Nrf2 pathways *in vitro*. The CCK-8 assay was used to determine the cell viability of HK-2 cells after 24 h treat with ADR **(A)**, ASIV **(B)**, Fer-1 **(C)**, and RSL3 **(D)**. HK-2 cells were treated with 1 µM ADR and 100 µM ASIV or 3 µM fer-1 **(E)** or 1 µM RSL3 **(F)** for 24 h. HK-2 cell viability was detected by the CCK-8 assay. The cellular ROS was detected after treatment **(G)**. Protein expression of ferroptosis-related proteins **(H,K)**, including GPx4 **(I,L)** and SLC7A11 **(J,M)** was used to detect after co-treatment of ADR and ASIV or fer-1. Data were expressed as mean ± SD, *n* = 6. *p < 0.05, **p < 0.01 vs. CON group. ^#^*p* < 0.05, ^##^*p* < 0.01 vs. ADR group.

To provide further evidence on ASIV protecting nephrocytes depending on modulating ferroptosis, HK-2 cells were treated with different concentrations of ADR (0.01–100 μM, Figure 5A), ASIV (6.25–200 μM, Figure 5B), Fer-1 (0.1–30 μM, Figure 5C), and RSL3 (0.1–10 μM, Figure 5D) for 24 h. The CCK-8 results revealed that the non-toxicity of ADR to HK-2 cells was at concentrations from 0.01 to 0.1 µM, ASIV at concentrations from 6.25 to 100 μM, Fer-1 at concentrations from 0.1 to 3 μM, and RSL3 at concentrations from 0.1 to 0.3 µM. Consistent with previous findings *in vivo*, our results demonstrated that ADR could inhibit HK-2 cells viability. However, the inhibitory effect of ADR was markedly elevated after either ASIV (100 µM) or Fer-1 (3 µM) co-treatment for 24 h (Figure 5E). Moreover, the alleviatory effect of ASIV on ADR-induced cell injury could be abrogated by RSL3 (1 µM) to some extent (Figure 5F). When the ROS of these cells were examined, we found ASIV inhibited ADR-induced ROS generation, which is similar to Fer-1. Meanwhile, the inhibitory effect of ASIV on ADR-induced ROS could be abrogated by RSL3 to some extent (Figure 5G). GPx4 is on the downstream of SLC7A11, utilizing GSH to detoxify lipid peroxides and prevent ferroptosis. Western blot showed that ASIV co-treatment increased the expression of GPx4 and SLC7A11 similar to Fer-1 when compared to the ADR treatment alone (Figures 5H–J). Furthermore, RSL3 partially inhibited the expression of GPx4 and SLC7A11 induced by ASIV when ADR, ASIV, and RSL3 co-treated HK-2 cells (Figures 5K–M). Collectively, our results demonstrated that ASIV might protect ADR-induced nephrocyte injury as a ferroptosis inhibitor.

### Astragaloside IV Inhibited Cellular Iron Uptake and Promoted Iron Efflux

**FIGURE 6:**
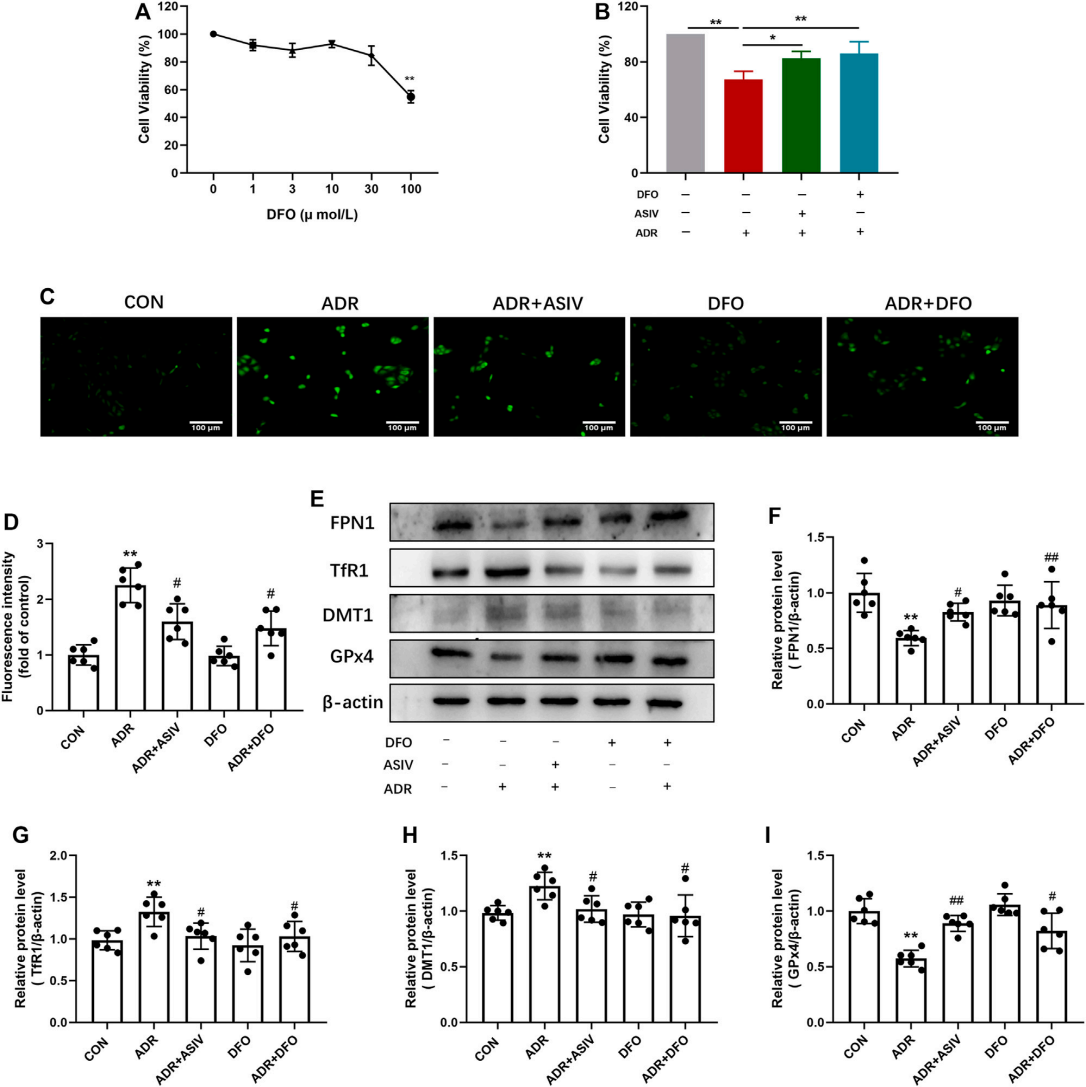
ASIV could alleviate ADR-induced cytotoxicity by reducing iron deposition. **(A)** After 24 h of DFO treatment, HK-2 cell viability was tested by the CCK-8 assay. **(B)** DFO 30μM + ADR 1 μM group for 24 h. HK-2 cell viability was evaluated by the CCK-8 assay. **(C)** The cellular ROS was detected after treatment and the chart. Representative image **(D)** and the protein levels of FPN1 **(E)**, TfR1 **(F)**, DMT1 **(G)**, and GPx4 **(H)** in the kidney tissue in these groups. Data were expressed as mean ± SD, *n* = 6. *p < 0.05, **p < 0.01 vs. CON group. ^#^*p* < 0.05, ^##^*p* < 0.01 vs. ADR group.

Iron plays an important role in mediating the production of reactive oxygen species and the enzyme activity of lipid peroxidation; thus, we detected proteins related to iron uptake and efflux. As shown in Figure 6A, DFO at concentrations of 1–30 µM had no obvious cytotoxicity to normal HK-2 cells; thus, 30 µM was chosen in the following experiment. There was significant elevation in cell viability after ASIV was treated compared to ADR alone, the effect is similar to that of DFO (Figure 6B). Consistent with this, ROS research has shown that ASIV treatment significantly decreased the production of ROS induced by ADR (Figure 6C). In comparison with the ADR group, DFO co-treatment resulted in a decrease in the ROS levels. Chelation mechanism may be involved to inhibit iron-mediated ROS production by Fenton reaction. However, no statistically significant difference was observed in the DFO-alone treatment group compared to the CON group. This may be attributed to the physical ROS level which is low in the CON group. The Western blot results show that ASIV lowered TfR1 and DMT-1 expression while enhancing the expression of FPN1 and GPx4 in ADR-administrated cells, the effects of which were akin to those of deferoxamine mesylate (Figures 6D–H).

### Astragaloside IV Activated the Nrf2 Signaling Pathway *via* Pi3K

**FIGURE 7:**
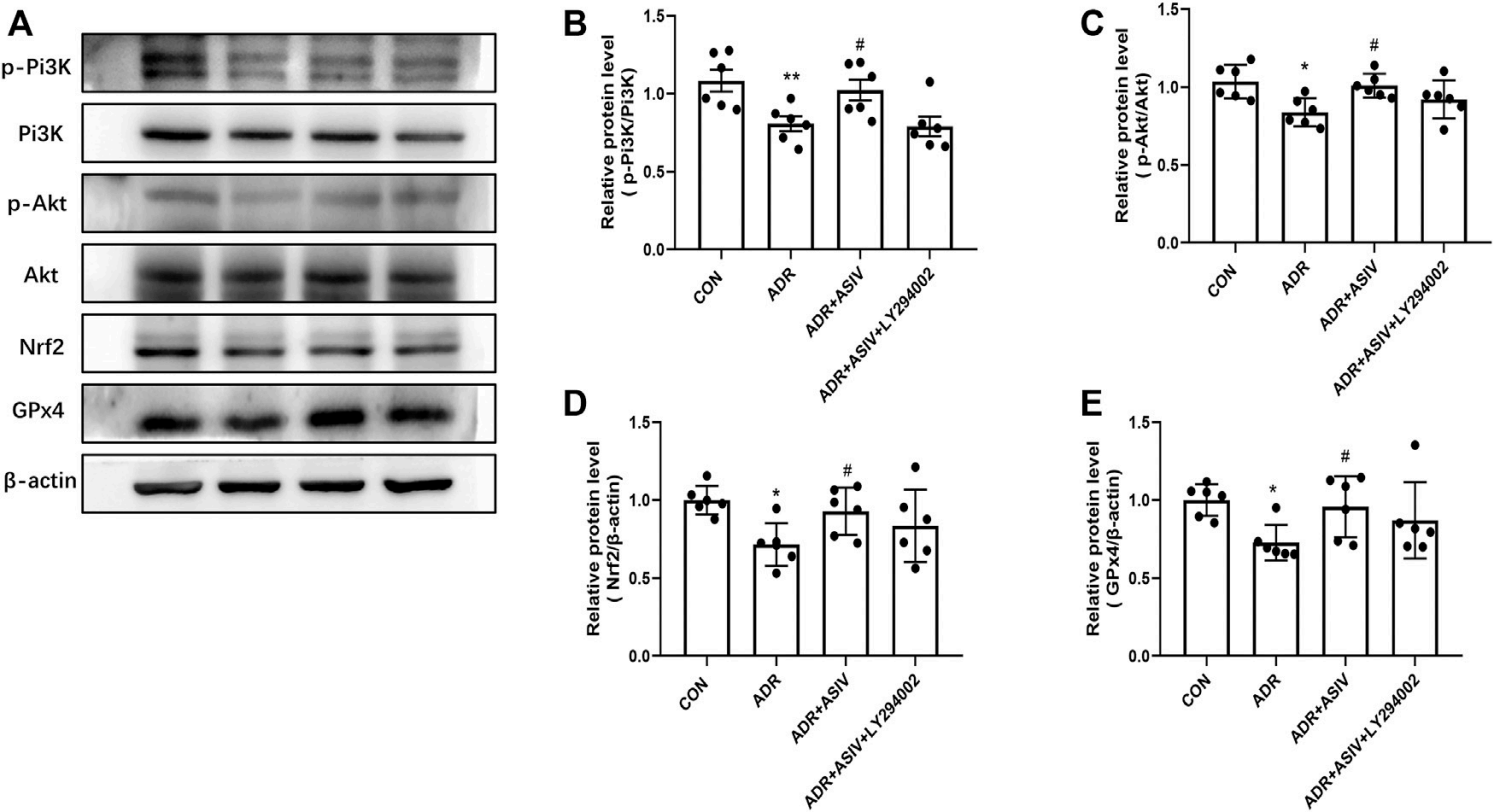
ASIV alleviated ADR-induced ferroptosis was related to the inhibition of the Pi3K/Akt/Nrf2 pathway *in vitro*. **(A)** Representative image for the levels of p-Pi3K, p-Akt, Nrf2, and GPx4 detected by Western blotting. Semiquantitative measurements of p-Pi3K **(B)**, p-Akt **(C)**, Nrf2 **(D)**, and GPx4 **(E)**. *p < 0.05, **p < 0.01 vs. CON group. Data were expressed as mean ± SD, *n* = 6. ^#^*p* < 0.05, ^##^*p* < 0.01 vs. ADR group.

Pi3K inhibitor LY294002 was used for analyzing how ASIV affected ferroptosis. Compared with ADR alone, ASIV co-treatment restored the expression levels of p-Pi3K, p-Akt, Nrf2, and GPx4 in HK-2 cells (Figures 7A–E). LY294002 has previously been shown to inhibit Pi3K, and co-treatment with LY294002 significantly inhibited the effect of ASIV on Pi3K (Figure 7B). Furthermore, LY294002 co-treated caused a reduced p-Akt/Akt ratio, as determined by p-Akt and Akt expression levels when compared to those of the ADR + ASIV group (Figure 7C). These results suggest that ASIV may protect ADR-induced nephrocyte injury through mediating the Pi3K/Akt signal pathway. Moreover, co-treated LY294002 decreased ASIV-induced Nrf2 expression in ADR-treated HK-2 cells, suggesting ASIV may enhance the Nrf2 pathway *via* the Pi3K/Akt pathway (Figure 7D). It is noteworthy that co-treated LY294002 abrogated the stimulatory effect of ASIV on the GPx4 activity to some extent but not fully (Figure 7E). The results mean that ASIV reactivated GPx4, at least partially, *via* PI3K.

## Discussion

Renal injury is one of the most common side effects that limit ADR for its application on antitumor therapy. Ferroptosis is known to be one form of non-apoptotic cell death and triggered by the iron-dependent accumulation of lipid ROS. We proposed that renal ferroptosis may be caused by ADR. Astragaloside IV (ASIV) is a kind of natural saponins involving anti-oxidation, anti-inflammatory, and anti-fibrosis. It is noteworthy that ASIV has been widely used in the treatment of kidney diseases, such as acute kidney injury (Yan et al., 2017) and diabetes mellitus kidney damage (Zhou et al., 2020). In this study, we found the renal cells in ADR-administrated rats showed the characteristics of ferroptosis such as shrunken mitochondria, elevated membrane density, and disappeared mitochondria ridge. Meanwhile, ASIV significantly ameliorated kidney injury, improved renal dysfunction, reduced oxidative stress, alleviated iron accumulation, and inhibited the induction of ferroptosis by ADR. ASIV also rescued the intracellular levels of Nrf2 and promoted nuclear translocation of Nrf2. *In vitro*, treatment of the HK-2 cells with fer-1 or DFO obviously improved cell viability during Adriamycin administration. On the other hand, the protective role of ASIV can be abrogated by RSL3 to some extent. Moreover, ASIV increased the phosphorylation of Pi3K, Akt, and the expression of Nrf2 and GPx4 compared to the HK-2 cells stimulated by ADR. However, Pi3K inhibitor LY294002 abrogated these activations. In conclusion, ferroptosis may be involved in ADR-induced nephrotoxicity; ASIV might protect nephrocytes against ADR-induced ferroptosis *via* activations of the Pi3K/AKT and Nrf2 signaling pathways.

ASIV was confirmed to alleviate ADR-induced cardiomyopathy by inhibiting oxidative stress. ASIV has also been reported to prevent iron overload-induced hepatic injury (Xie et al., 2019). Moreover, ASIV treatment is beneficial to rebalance the levels of oxidative stress in the placenta of PE rats by restoring the Nrf2/HO-1 pathway (Yang et al., 2020). Based on the research described earlier, we proposed that ASIV had beneficial effects on renal injury through inhibiting oxidative stress. The fluorescence of the DHE oxidation product increased in the ADR group and decreased when ASIV was co-treated. MDA, the final product of lipid peroxidation, is generally accepted as an oxidative stress marker. What is more, MDA is a contributing factor for ferroptosis activation. The MDA levels and the antioxidant enzymes activities of SOD and GPx have a concurrent variation with ROS, suggesting ADR may induce lipid peroxidation and ASIV has a significant role in MDA prevention closely related to activate antioxidant enzymes.

Both the accumulation of lipid peroxidation and reactive oxygen species (ROS) can attribute to iron; the regulators involved in iron metabolism like iron efflux, utilization, storage, and uptake-related proteins strictly controlled ferroptosis. The Perls’ Prussian blue staining showed that ASIV can decrease the accumulation of iron in renal tissues, and these results demonstrated that ASIV could protect the kidney against iron overload. DMT1 is a key iron transporter and contributes non-heme iron uptake. Moreover, TfR1 is in charge of the uptake of cellular iron and is supported as a biomarker for ferroptosis (Feng et al., 2020). For example, TfR1 knockdown attenuated erastin-induced ferroptotic cell death in RAS mutation cells (Yang and Stockwell, 2008). FPN1 is the only known intracellular non-heme iron effluxer. FPN1 knockdown accelerated erastin-induced ferroptosis through the accumulation of lipid ROS in neuroblastoma cells (Geng et al., 2018). Iron in the cytoplasm is stored by subunits of heavy (FTH) and light (FTL) ferritin chains. In addition, FTH exerts a key role in ferroptosis. FTH is involved in ferritinophagy resulting in the iron release, which leads to ferroptosis (Latunde–Dada, 2017). Moreover, our previous study revealed that ASIV exerts cardioprotective effects by inhibiting excessive autophagy induced by ADR (Luo et al., 2021b). Based on our results, ASIV decreased the accumulation of iron in the ADR-treated group. Furthermore, DFO inhibited iron accumulation in the ADR group. These results suggested that ASIV may alleviate kidney injury induced by ADR by decreasing iron accumulation.

Iron-dependent oxidative damage is a character of ferroptosis. In this study, we found ADR induced ferroptosis in renal cells by TEM and qPCR. ASIV treatment inhibited the ultrastructural and ptgs2 mRNA-level changes. Intriguingly, DFO and Fer-1 could reverse ADR-induced cell death, suggesting that ferroptosis exerts an important role in ADR-induced kidney damage. Furthermore, RSL3 and ASIV co-treatment partially inhibited the protective effect of ASIV on ADR-induced kidney injury, implying ASIV may decrease kidney injury by attenuating ferroptosis. SLC7A11 functions to import cystine into the cell, which is subsequently converted to the major cellular antioxidant GSH. Then, GPx4 utilizes GSH as a substrate to reduce PLOOH to lipid alcohols during ferroptosis. The compounds that inhibited GPx4 can initiate ferroptosis and lead to the accumulation of toxic lipid ROS (Forcina and Dixon, 2019). Based on our data, the decrease in GPx4 and SLC7A11 levels induced by ADR was alleviated by ASIV. However, the protective role of ASIV can be abrogated by RSL3 to some extent in HK2 cells. These results suggested that ASIV could protect against ADR-induced renal injury at least in part through mediating ferroptosis.

Nrf2, an oxidative stress-induced transcription factor, has been reported to exert a key role in regulating ferroptosis (Dodson et al., 2019). In addition, precious reports have shown that the activation of the Pi3K/Akt signal can trigger the nuclear translocation of Nrf2, thereby inhibiting reactive oxygen species and promoting cell survival. In this study, we determined whether ASIV can mediate Nrf2 activation *via* the activation of the Pi3K/Akt pathway. Our results showed that ASIV activated the Pi3K/Akt and Nrf2 signaling pathways in the kidney and HK-2 cells when compared with the ADR-alone group. Interestingly, when Pi3K was blocked by ly294002, the activation of Nrf2 was reversed. These data indicated that ASIV activated Nrf2 *via* the Pi3K/Akt signaling pathway, thereby exerting its anti-ferroptotic effects.

In summary, our results suggested that renal ferroptosis exerts a key role in the progression of ADR-induced renal injury and ASIV might protect nephrocytes against ADR-induced ferroptosis *via* activations of the Pi3K/Akt and Nrf2 signaling pathways.

## Data Availability

The original contributions presented in the study are included in the article/Supplementary Material, further inquiries can be directed to the corresponding authors.

## Abbreviations

ADR, Adriamycin; ASIV, astragaloside IV; CCK-8, Cell Counting Kit-8 Assay; DFO, deferoxamine mesylate; DMT1, divalent metal (ion) transporter 1; Fer-1, ferrostatin-1; FPN1, ferroportin 1; FTL, ferritin light chain; GPx, glutathione peroxidase; GPx4, glutathione peroxidase 4; GSH, glutathione; HE, hematoxylin and eosin; HK-2, the human proximal tubular epithelial cells; HO-1, heme oxygenase-1; MDA, malondialdehyde; MT, Masson’s trichrome; Nrf2, nuclear factor erythroid-2-related factor 2; PBS, phosphate-buffered saline; ROS, reactive oxygen species; SLC7A11, solute carrier family 7 member 11; TfR-1, transferrin receptor 1; 4-HNE, 4-hydroxynonenal.
